# Cyclic tensile strain affects the response of human periodontal ligament stromal cells to tumor necrosis factor-α

**DOI:** 10.1007/s00784-021-04039-8

**Published:** 2021-06-29

**Authors:** Zhongqi Zhao, Christian Behm, Marco Aoqi Rausch, Zhiwei Tian, Xiaohui Rausch-Fan, Oleh Andrukhov

**Affiliations:** 1 Competence Center for Periodontal Research, University Clinic of Dentistry and Periodontology, Medical University of Vienna, Sensengasse 2A, 1090 Vienna, Austria; 2 Division of Orthodontics, University Clinic of Dentistry, Medical University of Vienna, 1090 Vienna, Austria; 3 Center for Clinical Research, University Clinic of Dentistry, Medical University of Vienna, 1090 Vienna, Austria

**Keywords:** Human periodontal ligament stromal cells, Orthodontic force, Mechanical loading, Periodontitis, Inflammatory cytokine

## Abstract

**Objectives:**

Orthodontic treatment in adult patients predisposed to mild or severe periodontal disease is challenging for orthodontists. Orthodontic malpractice or hyper-occlusal forces may aggravate periodontitis-induced destruction of periodontal tissues, but the specific mechanism remains unknown. In the present study, the combined effect of mechanical stress and tumor necrosis factor (TNF)-α on the inflammatory response in human periodontal ligament stromal cells (hPDLSCs) was investigated.

**Materials and methods:**

hPDLSCs from 5 healthy donors were treated with TNF-α and/or subjected to cyclic tensile strain (CTS) of 6% or 12% elongation with 0.1 Hz for 6- and 24 h. The gene expression of interleukin (IL)-6, IL-8 and cell adhesion molecules VCAM and ICAM was analyzed by qPCR. The protein levels of IL-6 and IL-8 in conditioned media was measured by ELISA. The surface expression of VCAM-1 and ICAM-1 was quantified by immunostaining followed by flow cytometry analysis.

**Results:**

TNF-α-induced IL-6 gene and protein expression was inhibited by CTS, whereas TNF-α-induced IL-8 expression was decreased at mRNA expression level but enhanced at the protein level in a magnitude-dependent manner. CTS downregulated the gene expression of VCAM-1 and ICAM-1 under TNF-α stimulation, but the downregulation of the surface expression analyzed by flow cytometry was observed chiefly for VCAM-1.

**Conclusions:**

Our findings show that mechanical force differentially regulates TNF-α-induced expression of inflammatory mediators and adhesion molecules at the early stage of force application. The effect of cyclic tensile strain is complex and could be either anti-inflammatory or pro-inflammatory depending on the type of pro-inflammatory mediators and force magnitude.

**Clinical relevance:**

Orthodontic forces regulate the inflammatory mediators of periodontitis. The underlying mechanism may have significant implications for future strategies of combined periodontal and orthodontic treatment.

**Supplementary Information:**

The online version contains supplementary material available at 10.1007/s00784-021-04039-8.

## Introduction

Periodontal ligament (PDL) is a connective tissue between the cementum and the alveolar bone, which supports the teeth and is continuously subjected to and responding to varied types of biomechanical forces [1, 2]. During orthodontic treatment, appropriate mechanical force squeezes or stretches the PDL, regulating a coordinated remodeling process which consists of bone resorption at compression side and bone formation at tension side of the alveolar bone, and culminating in the orthodontic tooth movement (OTM) [3]. This process highly depends on the cellular components of PDL, which principally consist of undifferentiated resident mesenchymal stromal cells (MSCs), fibroblasts and osteoblasts [1]. Human periodontal ligament stromal cells (hPDLSCs), the resident MSCs, are a heterogeneous cell population and have a fibroblast-like morphology [4, 5]. They are multipotent progenitor cells expressing specific MSC surface markers, displaying multi-lineage differentiation potential [5, 6] and exerting immunomodulatory effects [7]. hPDLSCs are reported to be sensitive to mechanical loading and play a central role in bone remodeling during OTM [8].

Currently, an increasing number of adult patients predisposed to mild or severe periodontal disease are seeking orthodontic treatment, leading to the challenges of maintaining homeostasis of the periodontium which orthodontists have to face [9]. It is known that orthodontic malpractice or hyper-occlusal forces may aggravate periodontitis-induced destruction of periodontal tissues [10]. The periodontal tissue of periodontitis patients is considered to be more sensitive and less tolerant to mechanical loading than that in healthy individuals [11–14] but the underlying mechanisms for the co-destructive effect are not yet clear. Tumor necrosis factor-α (TNF-α) is a primary and initial inflammatory cytokine which is highly correlated with the pathogenesis of periodontitis [15, 16]. TNF-α up-regulates the expression of numerous inflammatory mediators in hPDLSCs, particularly IL-6, IL-8, vascular cell adhesion molecule 1 (VCAM-1), and intercellular adhesion molecule 1 (ICAM-1) [16–18]. IL-6 is closely related to bone destruction through its effects on osteoclastogenesis [19, 20]; IL-8 plays a key role in the recruitment and activation of neutrophils to the site of tissue damage or infection [21]. VCAM-1 and ICAM-1 stimulate leukocyte recruitment required for inflammation [18]. Furthermore, ICAM-1 and VCAM-1 mediate the immunomodulatory function of MSCs [22].

Orthodontic treatment also stimulates various inflammatory mediators in response to mechanical forces. During orthodontic tooth movement, the ongoing remodeling process begins with an initial aseptic inflammatory response [23]. This is characterized by production of numerous mediators like IL-1β, IL-6, IL-8, and TNF-α [11, 24–27], which are also involved in the progression of periodontitis. Therefore, interference between the biological mediators induced by mechanical stimulation and periodontal inflammation might contribute to the co-destructive effect, which is not always confirmed by experimental data. Qualitatively, the impact of orthodontic forces on the inflammatory response seems to depend on force magnitude [9, 28]. On the one hand, in vitro studies on human PDL cells reported that mechanical forces with the magnitude of 12–20% aggravated inflammatory response induced by bacterial components [29, 30]. Furthermore, an in vivo study reported that orthodontic force up-regulated the expression of IL-1β and TNF-α in periodontitis rats and amplified bone loss [11]. On the other hand, a number of studies have shown that low magnitude-mechanical forces with magnitude up to 10% can also exert anti-inflammatory effects in human PDL cells [28, 31, 32].

Hence, the impact of mechanical load to the inflammatory response should be still investigated. Of particular interest is the question how the mechanical load might influence the response of hPDLSCs to the inflammatory environment. Although TNF-α is involved in both periodontitis and orthodontic tooth movement, the combined effect of TNF-α and mechanical strain on the inflammatory responses was never investigated in hPDLSCs to date. Therefore, the aim of the present in vitro study was to investigate the effects of CTS of different magnitudes on TNF-α-induced inflammatory response in hPDLSCs. CTS with either 6% or 12% elongation was applied to identify the role of low and high magnitude orthodontic forces. Cells were stimulated for either 6 or 24 h, because these time points reflect the initial stage of applied mechanical forces during orthodontic treatment [33], at which the inflammatory processes play the most essential role [34]. The TNF-α-induced response was evaluated based on the resulting expression of IL-6, IL-8, VCAM-1, and ICAM-1.

## Materials and methods

### Cell culture

All the procedures were performed in accordance with the Declaration of Helsinki and the “Good Scientific Practice” guidelines of the Medical University of Vienna and approved by the Ethics Committee of the Medical University of Vienna (ethical approval number: 1079/2019, extended in 2019). Primary hPDLSCs of 5 periodontally healthy patients were isolated from the third molars extracted for orthodontic reasons. Periodontal ligaments were dissected from the middle third of the root surface and cultured in Dulbecco’s modified Eagles Medium (DMEM, Sigma-Aldrich, St. Louis, USA), supplemented with 10% fetal bovine serum (FBS, Gibco, Carlsbad, USA), 100 U/ml penicillin and 50 µg/ml streptomycin (P/S, Gibco, Carlsbad, CA, USA). Outgrowing cells were kept at 37 °C under an atmosphere of 95% humidity and 5% CO_2_. After reaching confluency, cells were detached with accutase (Sigma-Aldrich, St. Louis, USA) and passaged. Cells between passage 4–7 were used in all subsequent experiments. The phenotype of isolated hPDLSCs was verified according to our recent study [4]: cells were positively stained with mesenchymal cell markers (CD29, CD73, CD90, CD105, and CD146) and negatively stained with hematopoietic stem cell markers (CD31, CD34, and CD45), which is in agreement with the official criteria for MSCs from the International Society for Cell and Gene Therapy (ISCT) [35, 36]. The representative FACS dot plots are provided in Supplementary Fig. 1.

### Application of tensile strain and TNF-α treatment

**Fig. 1:**
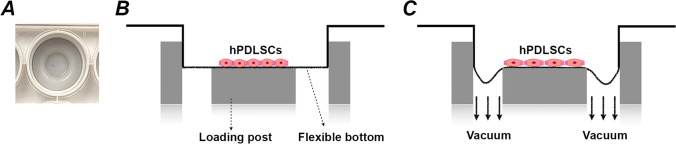
Schematic of mechanical force loading. (**a**) BioFlex® Cell Seeders were applied before seeding to confine the cell suspension in the central area of a well in the culture plates. (**b**) The culture plates were transferred onto the loading post after seeding hPDLSCs at a density of 1 × 10^5^ cells/well in the central region of the flexible membrane using BioFlex® Cell Seeders. (**c**) Cells were stretched by a cyclic tensile strain (equibiaxial, 6 or 12% elongation, sinusoidal curve, 0.1 Hz, 6 or 24 h) when the vacuum was applied

hPDLSCs were seeded onto collagen type I-coated BioFlex® six-well culture plates (Flexcell® International Corporation, Burlington, NC, USA) at the initial density of 1 × 10^5^ cells/well in 3 ml of DMEM with all supplements. BioFlex® Cell Seeders (Flexcell® International Corporation, Burlington, NC, USA) were used during plating to confine cells to the central region of the flexible membrane. After one-day incubation in supplemented DMEM cells reached 80–95% confluence and were serum-starved for 24 h before the further treatment. The confluent cells were used for the experiments, undergoing inflammatory and/or mechanical stimulation. To mimic a pro-inflammatory microenvironment, hPDLSCs were treated with 10 ng/ml TNF-α (Invivogen, San Diego, USA) for 6- or 24 h. To simulate mechanical forces at tension sites, hPDLSCs were subjected to a cyclic tensile strain (CTS, 6 or 12% elongation, sinusoidal curve, 0.1 Hz, 6 or 24 h) using a Flexcell® FX-5000™ Tension System (Flexcell® International Corporation, Burlington, NC, USA). The plates were placed on a loading station on which the vacuum stretched the flexible silicon membrane, causing deformation along the loading post-surface and thereby generating the dynamic equibiaxial tensile strain to attached cells (Fig. 1). The parameters of stretching were chosen to be within the physiological range based on the existing literature [28, 37–40]. Untreated cells were maintained under the same conditions but without mechanical stimulation.

### Reverse transcription-quantitative polymerase chain reaction (RT-qPCR)

After 6- and 24 h of incubation, hPDLSCs were lysed and mRNA was transcribed into cDNA using TaqMan Gene expression Cells-to-CT kit (Applied Biosystems, Foster City, USA) following the manufacturer’s instruction. Reverse transcription was performed using Primus 96 advanced thermocycler (Peq/Lab/VWR, Darmstadt, Germany) with the following settings: 37 °C for 1 h and 95 °C for 5 min followed by 4 °C. Quantitative analysis of the gene expression was done using a QuantStudio 3 device (Applied Biosystems, Foster City, USA). The following TaqMan Gene Expression Assays (Applied Biosystems, Foster City, USA) were used with following ID numbers: IL-6, Hs00985639_m1; IL-8, Hs00174103_m1; VCAM-1, Hs00365486_m1; ICAM-1, Hs00164932_m1; and glyceraldehyde-3-phosphate dehydrogenase (GAPDH), Hs99999905_m1. The reaction was performed by heated at 95 °C for 10 min and then followed by 50 cycles, each cycle consisting of denaturation at 95 °C for 15 s and annealing/extension at 60 °C for 1 min. The expression of target genes was calculated using 2^−ΔΔCt^ method, using GAPDH as the housekeeping gene and untreated cells as a control [41].

### Enzyme-linked immunosorbent assay

After 6- and 24 h of incubation, conditioned media were harvested and protein levels of IL-6 and IL-8 were measured using Human Uncoated IL-6 ELISA and Human Uncoated IL-8 ELISA (both from ThermoFisher Scientific, Waltham, USA) according to the manufacturer’s instruction. The optical density (OD) was measured at 450 nm and 570 nm using a Synergy HTX multi-mode reader (BioTek Instruments, Winooski, USA). After subtracting OD_570_ from OD_450_, final concentrations were calculated by plotting determined OD values against the appropriate standard curve using Gen5 All-In-One Microplate Reader Software version 2.09 (BioTek Instruments, Winooski, USA). The detection limit was 2 pg/ml for both IL-6 and IL-8. All values below the detection limit were considered as zero for the analysis.

### Flow cytometry

After 6- and 24 h of incubation, the surface expression of VCAM-1 and ICAM-1 was measured by flow cytometry similarly to previously described methods [42]. Briefly, cells were harvested, resuspended in FACS buffer (PBS supplemented with 3% BSA and 0.09% of NaN_3_) and fixed with 2% (v/v) formaldehyde (Merck KGaA, Darmstadt, Germany) for 30 min at room temperature. After washing and resuspending hPDLSCs in FACS buffer, the cells were stained 1:50 with phycoerythrin (PE)-conjugated mouse anti-human CD106 (VCAM-1) antibody or CD54 (ICAM-1) antibody (both from eBioscience, San Diego, USA) for 30 min in the dark at room temperature. Following washing single-cell suspensions were prepared in 400 μl FACS buffer. Surface marker expression was analyzed using the Attune NxT Acoustic Focusing Cytometer (ThermoFisher Scientific, Waltham, USA). Unstained hPDLSCs were used as control to define the positive threshold and adjust the instrument settings. The percentage of VCAM-1 or ICAM-1 positive hPDLSCs and the corresponding mean fluorescence intensities (MFI) were determined using Attune NxT software version 3.1.2 (ThermoFisher Scientific, Waltham, USA). In total, 10.000 cells were acquired per sample.

### Statistical analysis

All measured data were analyzed using SPSS 20.0 software (IBM, Armonk, USA). Normal distribution was proved by Kolmogorov–Smirnov test. Differences between groups were assessed by Wilcoxon signed-rank test, and paired-samples Student’s t-test. *p* values < 0.05 were considered significant. Data are presented as mean ± standard deviation of at least 5 independent repetitions with hPDLSCs isolated from at least 5 different individuals. Each experiment was performed with technical duplicates.

## Results

### Effects of TNF-α on IL-6 and IL-8 gene and protein expression

**Fig. 2:**
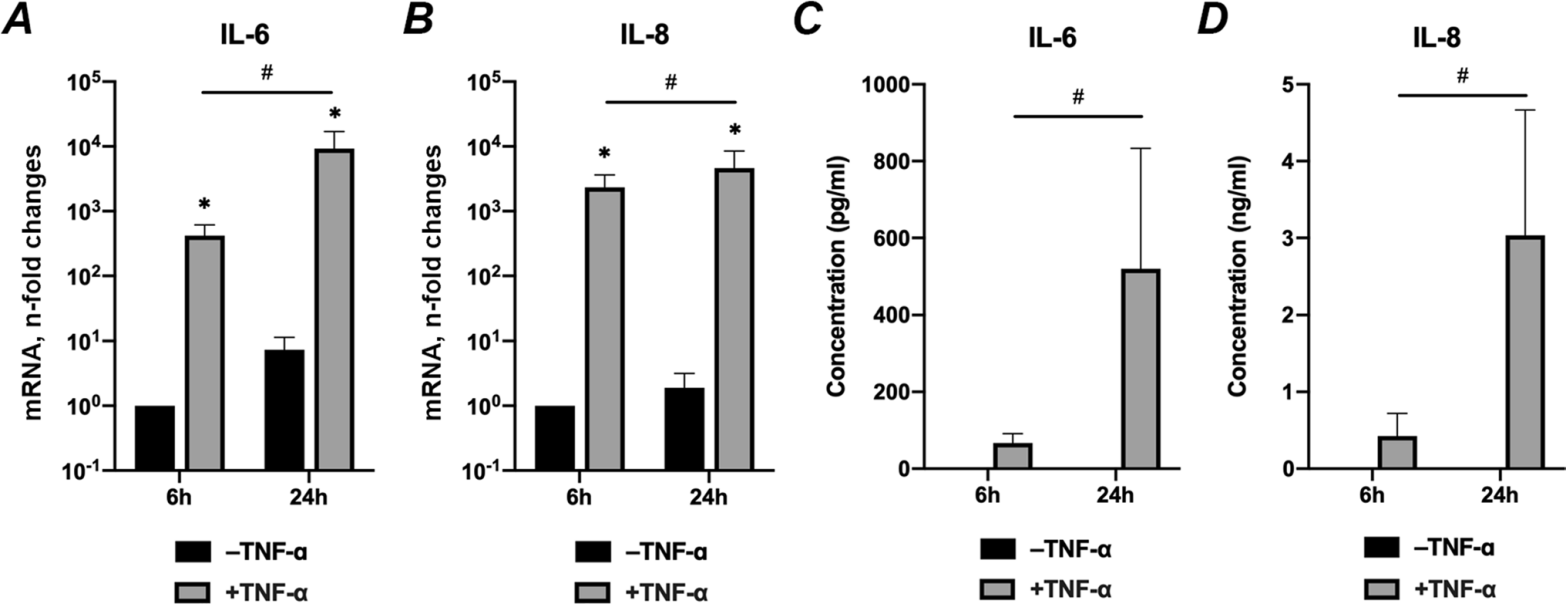
Effects of TNF-α on gene and protein expression of IL-6 and IL-8 on unstretched hPDLSCs. Primary hPDLSCs were treated with 10 ng/ml TNF-α without mechanical stretching. After 6- or 24 h of incubation, gene expression levels of IL-6 (**a**), IL-8 (**b**) were measured using RT-qPCR. The Y-axes show the n-fold changes in mRNA expression compared to untreated control after 6 h (n-fold expression = 1). The corresponding protein levels of IL-6 (**c**) and IL-8 (**d**) in conditioned media were quantified by ELISA. The groups in the absence or presence of TNF-α are indicated as -TNF-α or + TNF-α, respectively. The data are presented as the mean ± standard deviation. *p < 0.05 compared to corresponding control. ^#^*p* < 0.01 compared between groups as indicated

Figure 2 shows the effect of TNF-α on IL-6 and IL-8 expression in hPDLSCs. Exposure of hPDLSCs to TNF-α enhanced gene expression of IL-6 and IL-8 after both 6- and 24 h (Figs. 2a and 2b). TNF-α-induced gene expression of IL-6 and IL-8 was significantly increased with time. In the absence of TNF-α, IL-6 and IL-8 were below ELISA’s detection limit. After 6 h incubation in the presence of TNF-α, IL-6 was below detection limit in some samples. TNF-α induced the protein production of both IL-6 and IL-8, which was significantly increased in a time-dependent manner (Figs. 2c and 2d).

### Effects of CTS on IL-6 expression in the absence and presence of TNF-α

**Fig. 3:**
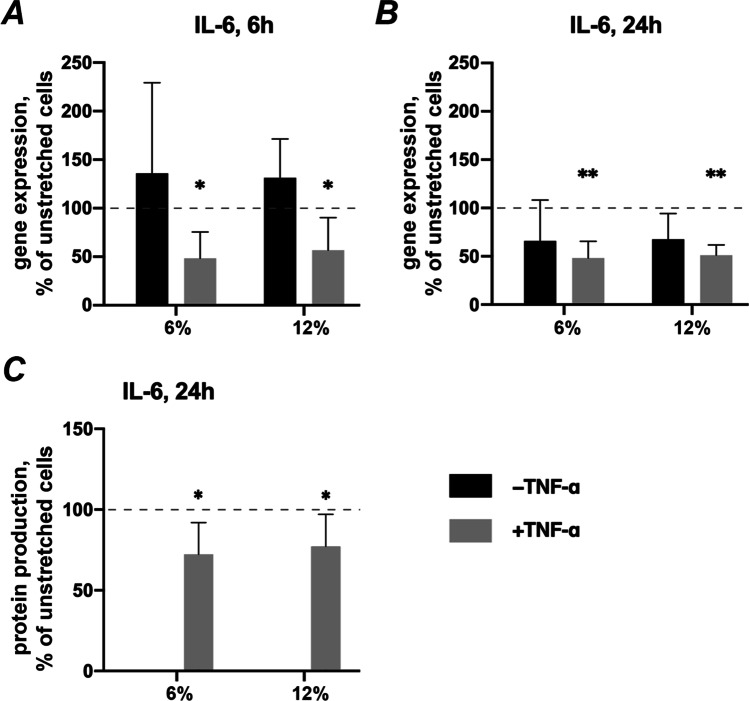
Effects of CTS on IL-6 expression in the absence / presence of TNF-α in hPDLSCs. Primary hPDLSCs were subjected to either 6% or 12% cyclic stretching, in the absence or presence of 10 ng/ml TNF-α for 6 or 24 h. IL-6 gene expression levels were measured with RT-qPCR after 6 (**a**) or 24 (**b**) hours. TNF-α-induced IL-6 protein levels after 24 h (**c**) were quantified by ELISA. Y-axes show the effect of CTS on IL-6 expression as % of the values observed in unstretched cells with or without TNF-α. For RT-qPCR (**a**, **b**), n-fold gene expression was calculated first using the 2^−ΔΔCt^ method and then the data were normalized to those observed in unstretched cells (100%, dashed line). For ELISA (**c**), the values were calculated as % of the values measured in unstretched cells (100%, dashed line). The data are presented as the mean ± standard deviation. * and ** — significantly different compared to unstretched control with *p* < 0.05 and *p* < 0.01, respectively

The effect of CTS on basal and TNF-α induced IL-6 expression in hPDLSCs is shown in Fig. 3. Application of CTS in the absence of TNF-α had no significant effect on the gene expression of IL-6 in hPDLSCs compared to the unstretched control groups (Fig. 3a and 3b). In contrast, TNF-α-induced IL-6 expression was inhibited by CTS at both magnitudes. A significant effect of CTS on TNF-α-induced IL-6 gene expression was observed after both 6- and 24 h (Figs. 3a and 3b, respectively). After 24 h, TNF-α-induced IL-6 protein production was significantly decreased by CTS with both 6% and 12% elongation (Fig. 3c). The relative IL-6 protein levels were not calculated for 6 h stimulation, because TNF-α-induced IL-6 protein production was below the detection limit in some samples independently on the mechanical stimulation.

### Effects of CTS on IL-8 expression in the absence and presence of TNF-α

**Fig. 4:**
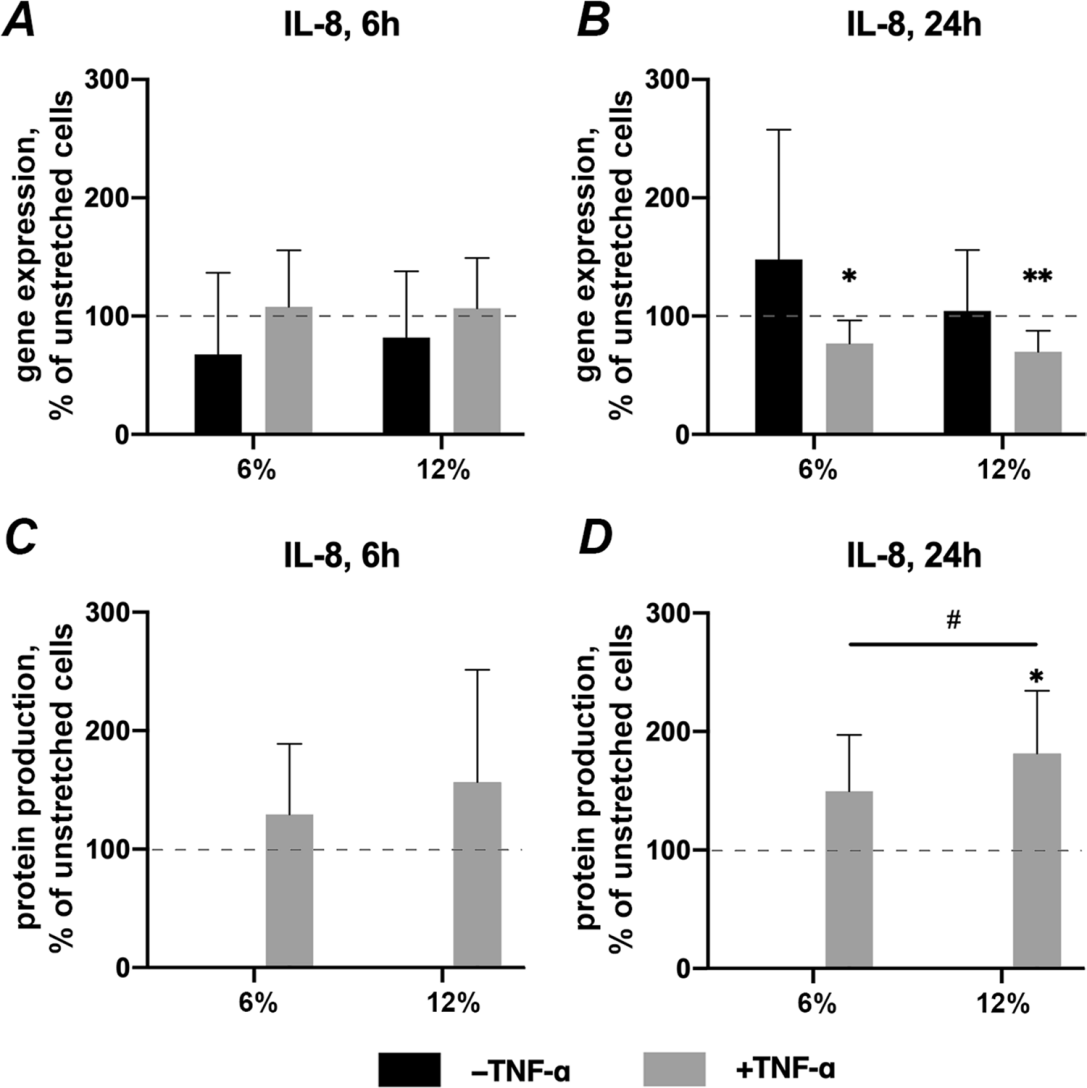
Effects of CTS on IL-8 expression in the absence / presence of TNF-α in hPDLSCs. Primary hPDLSCs were subjected to either 6% or 12% cyclic stretching, in the absence or presence of 10 ng/ml TNF-α. IL-8 gene expression levels were measured with RT-qPCR after 6 (**a**) or 24 (**b**) hours. TNF-α-induced IL-8 protein levels after 6 (**c**) and 24 (**d**) hours were quantified by ELISA. Y-axes show the effect of CTS on IL-8 expression as % of the values observed in unstretched cells with or without TNF-α. For RT-qPCR (**a**, **b**), n-fold gene expression was calculated first using the 2^−ΔΔCt^ method and then the data were normalized to those observed in unstretched cells (dashed line). For ELISA (**c**, **d**), the values were calculated as % of the values measured in unstretched cells (100%, dashed line). The data are presented as the mean ± standard deviation. * and ** — significantly different compared to unstretched control with *p* < 0.05 and *p* < 0.01, respectively

Figure 4 shows the effect of CTS on basal and TNF-α induced IL-8 expression in hPDLSCs. CTS applied on hPDLSCs did not influence the IL-8 gene expression level in the absence of TNF-α after both 6- and 24 h (Figs. 4a and 4b, respectively). Compared to unstretched hPDLSCs, TNF-α-induced IL-8 gene expression was not affected by CTS after 6 h (Fig. 3a). After 24 h incubation, CTS significantly decreased TNF-α-induced IL-8 expression using both, 6 and 12% elongation (Fig. 3b). In contrast to gene expression, TNF-α-induced IL-8 protein level was significantly increased by CTS in a dose-dependent manner after 24 h (Fig. 4d). A similar tendency was also observed after 6 h but without any statistical significance (Fig. 4c).

### Effects of TNF-α on VCAM-1 and ICAM-1 gene and protein expression

**Fig. 5:**
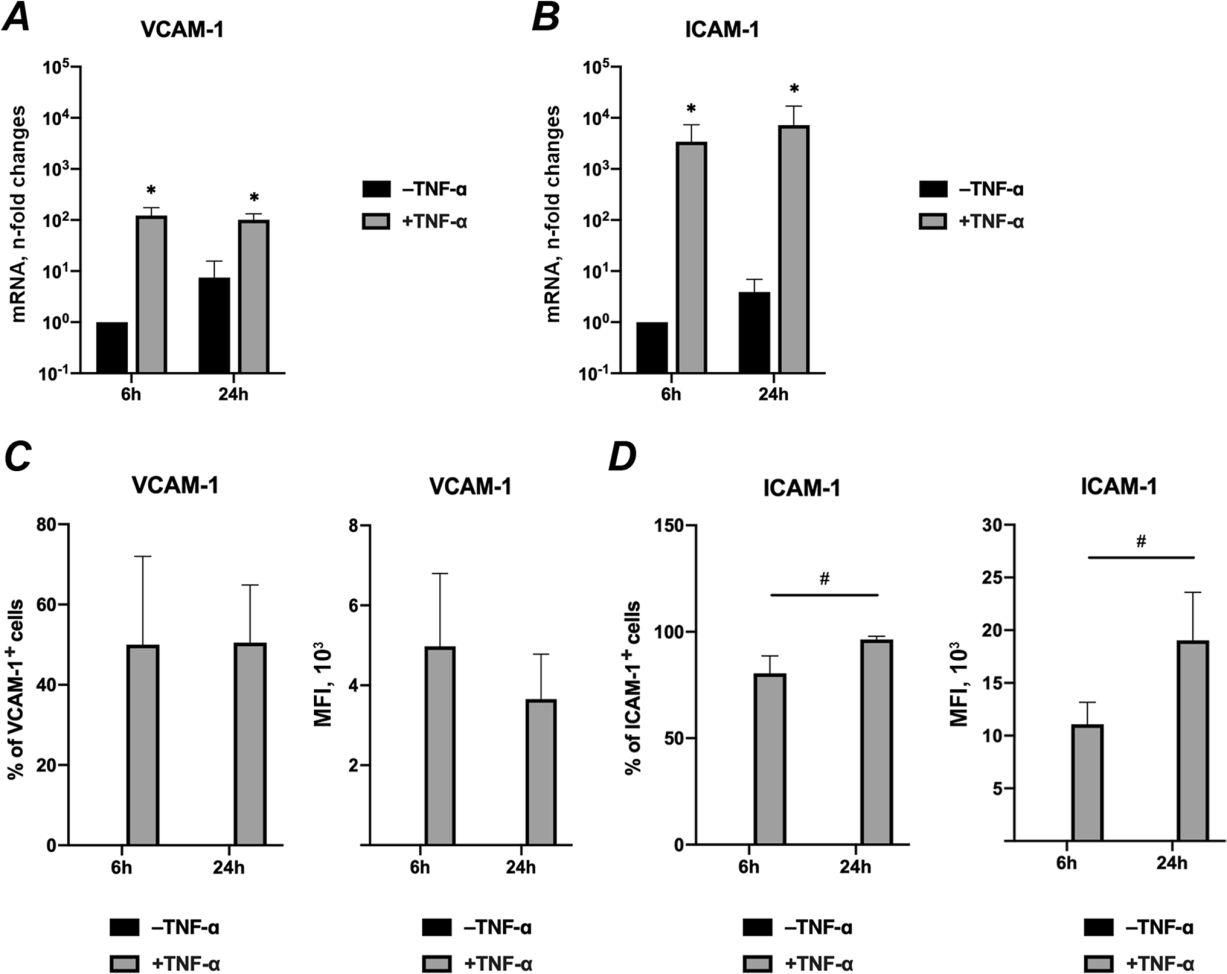
Effects of TNF-α on gene and protein expression of VCAM-1 and ICAM-1 in unstretched hPDLSCs. Primary hPDLSCs were treated with 10 ng/ml TNF-α without mechanical stretching. The groups in the absence or presence of TNF-α are indicated as -TNF-α or + TNF-α, respectively. After 6- or 24 h of incubation, gene expression levels of VCAM-1 (**a**) and ICAM-1 (**b**) were measured using RT-qPCR. The Y-axes show the n-fold changes in mRNA expression compared to untreated control after 6 h (n-fold expression = 1). The corresponding protein levels of VCAM-1 (**c**) and ICAM-1 (**d**) were quantified by flow cytometry. The Y-axes show the percentage of positive cells and the corresponding mean fluorescence intensity (MFI) of the positive cell population, respectively. No positively stained VCAM-1 and ICAM-1 cells were observed in the absence of TNF-α. The data are presented as the mean ± standard deviation. *p < 0.05 compared to corresponding control. ^#^*p* < 0.01 compared between groups as indicated

Figure 5 shows the effect of TNF-α on VCAM-1 and ICAM-1 expression in hPDLSCs. Exposure of hPDLSCs to TNF-α significantly enhanced gene and protein expression of VCAM-1 and ICAM-1 after both 6- and 24 h. TNF-α-induced protein expression of ICAM-1 was significantly increased with time (Fig. 5d). In the absence of TNF-α, hPDLSCs showed no VCAM-1 or ICAM-1 positive cell population.

### Effects of CTS on VCAM-1 gene expression and surface protein production in the absence and presence of TNF-α

**Fig. 6:**
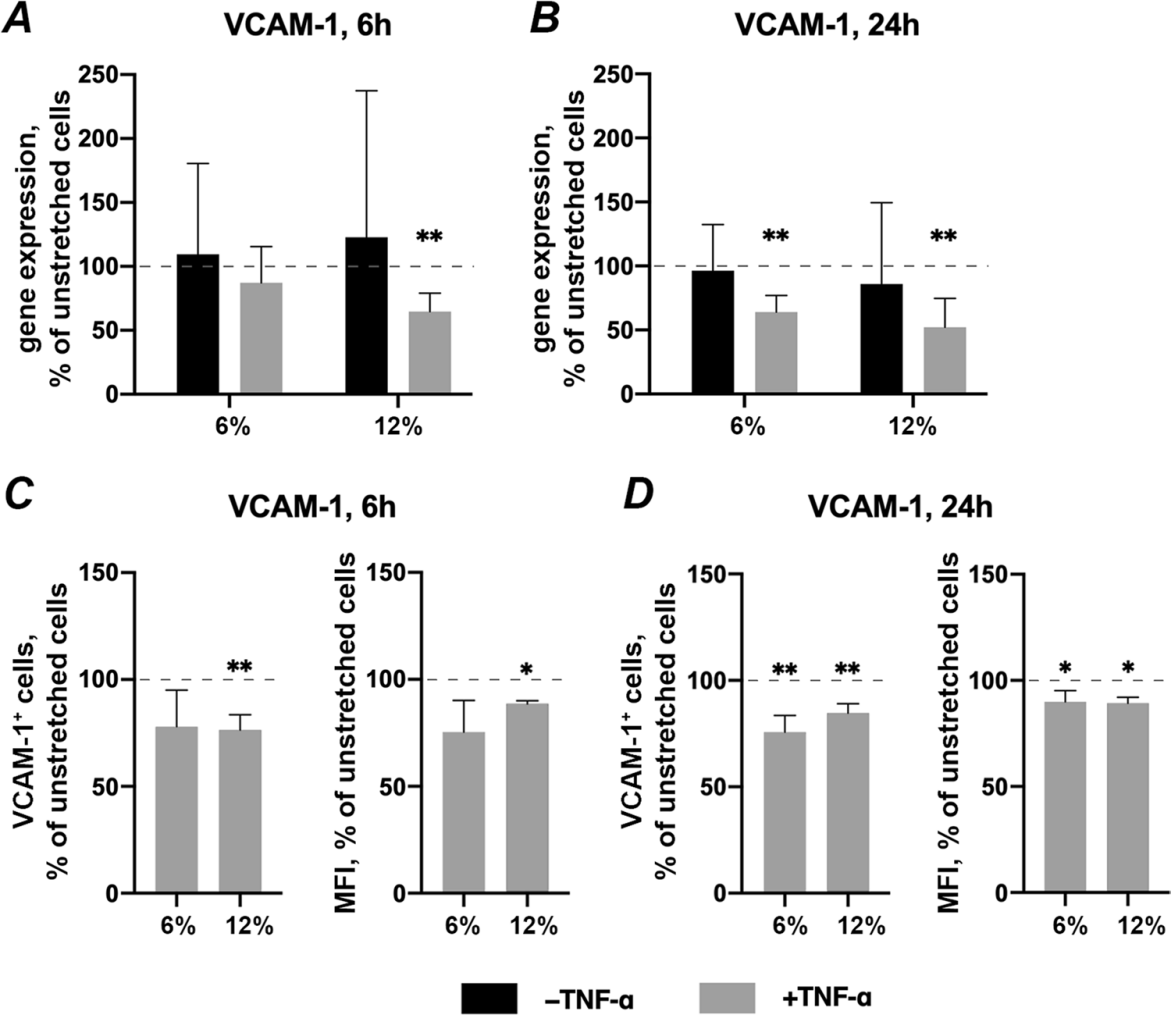
Effects of CTS on VCAM-1 expression in the absence/presence of TNF-α in hPDLSCs. Primary hPDLSCs were subjected to either 6% or 12% cyclic stretching, in the absence or presence of 10 ng/ml TNF-α. VCAM-1 gene expression levels were measured with RT-qPCR using the 2^−ΔΔCt^ method after 6 (**a**) or 24 (**b**) hours. TNF-α-induced VCAM-1 protein levels after 6 (**c**) and 24 (**d**) hours were quantified by flow cytometry. Y-axes show the effect of CTS on VCAM-1 expression as % of the values observed in unstretched cells with or without TNF-α. For RT-qPCR (**a**, **b**), n-fold gene expression was calculated first using the 2^−ΔΔCt^ method and then the data were normalized to those observed in unstretched cells (dashed line). For protein expression (**c**, **d**), Y-axes show the percentage of positive cells and the mean fluorescence intensity (MFI) of the positive cell population, in % of the values measured in unstretched cells. The data are presented as the mean ± standard deviation. * and ** — significantly different compared to unstretched control with *p* < 0.05 and *p* < 0.01, respectively

The effect of CTS on VCAM-1 expression in hPDLSCs under different experimental conditions is shown in Fig. 6. Application of CTS on hPDLSCs had no significant effect on the VCAM-1 gene expression level in the absence of TNF-α after 6- or 24 h (Figs. 6a and b). Compared to unstretched hPDLSCs, TNF-α-induced VCAM-1 gene expression was significantly downregulated by 12% CTS after 6 h (Fig. 6a) and by CTS at both magnitudes after 24 h (Fig. 6b). Qualitatively similar results were observed for TNF-α- induced VCAM-1 protein expression. The percentage of VCAM-1 positive cells and the MFI of the positive cell population was decreased by 12% CTS after 6 h (Fig. 6c) and decreased by CTS at both magnitudes after 24 h (Fig. 6d).

### Effects of CTS on ICAM-1 gene and surface protein expression in the absence and presence of TNF-α

**Fig. 7:**
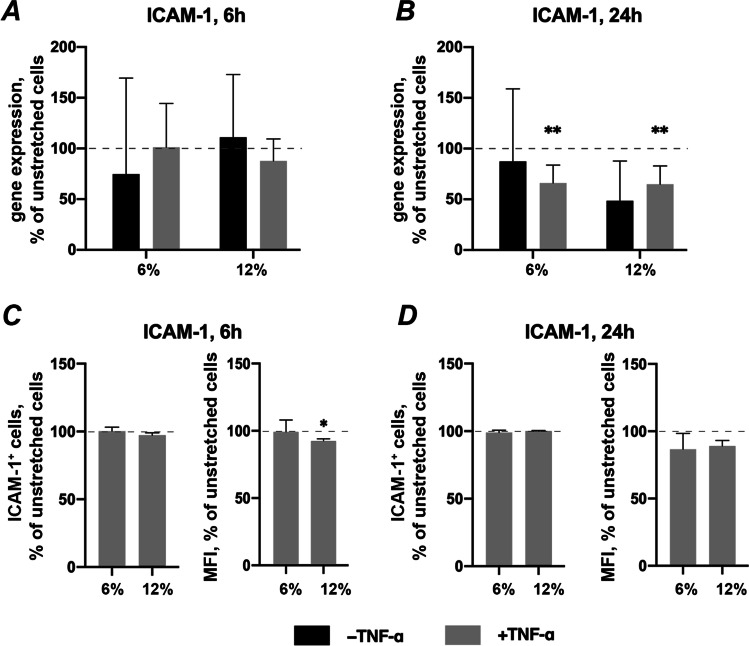
Effects of CTS on ICAM-1 expression in the absence / presence of TNF-α in hPDLSCs. Primary hPDLSCs were subjected to either 6% or 12% cyclic stretching, in the absence or presence of 10 ng/ml TNF-α. Unstretched hPDLSCs served as control. ICAM-1 gene expression levels were measured with RT-qPCR after 6 (**a**) or 24 (**b**) hours. TNF-α-induced ICAM-1 protein levels after 6 (**c**) and 24 (**d**) hours were quantified by flow cytometry. Y-axes show the effect of CTS on ICAM-1 expression as % of the values observed in unstretched cells with or without TNF-α. For RT-qPCR (**a**, **b**), n-fold gene expression was calculated first using the 2^−ΔΔCt^ method and then the data were normalized to those observed in unstretched cells (dashed line). For protein expression (**c**, **d**) the Y-axes show the percentage of positive cells and the mean fluorescence intensity (MFI) of the positive cell population, in % of the values measured in unstretched cells. The data are presented as the mean ± standard deviation. * and ** — significantly different compared to unstretched control with *p* < 0.05 and *p* < 0.01, respectively

Figure 7 shows the effect of CTS on basal and TNF-α induced ICAM-1 gene and protein expression in hPDLSCs. CTS applied on hPDLSCs did not influence the basal ICAM-1 gene expression level after both 6- and 24 h (Figs. 7a and 7b, respectively). Compared to unstretched hPDLSCs, TNF-α-induced ICAM-1 gene expression was not affected by CTS after 6 h (Fig. 7a) whereas it was downregulated by both 6% and 12% elongation after 24 h of incubation (Fig. 7b). The percentage of ICAM-1 positive cells induced by TNF-α was not affected by CTS (Figs. 7c and 7d). The MFI of the positive cell population was significantly decreased by 12% CTS after 6 h of incubation (Fig. 7c).

## Discussion

During orthodontic treatment, periodontal ligament (PDL) transmits the mechanical forces into biological cues through responding to immediate strain induced by extracellular matrix deformation [1, 43]. hPDLSCs play a central role in this process and participate in the adaptation of periodontal tissues to mechanical loading by mediating not only the self-renewing of the ligament but also the remodeling of alveolar bone [44, 45]. This process is initiated by an aseptic inflammatory response onsetting early several days after loading. Several in vitro studies demonstrated that CTS activates the production of numerous proinflammatory mediators [25, 46–49]. Moreover, application of CTS to human PDL cells in the presence of local periodontitis-related inflammation might aggravate the inflammatory processes [9, 29, 30, 50]. Therefore, the initial inflammation induced by orthodontic forces maybe involved in the clinically co-destructive effect of the mechanical forces and periodontitis-induced tissue destruction [29]. However, some in vitro reports also showed that CTS might have either pro- or anti-inflammatory effects depending on the magnitude [28, 29, 31, 51, 52]. Therefore, the underlying mechanism of the co-destructive effect remains unclear.

In the present study, we investigated for the first time the effects of CTS on the hPDLSCs response to TNF-α. CTS is commonly used in studies simulating orthodontic forces on the tension side in vitro [9, 30, 32, 39, 40, 50]. The physiological relevance of the cyclic strain is rather disputable. Some researchers considered that translation of the orthodontic force from an individual tooth onto the single-cell involves the static force [4, 33, 53, 54] Application of superelastic nickel titanium springs displayed a typical force plateau [55]. Nevertheless, the cyclic force still plays a predominant role in the in vitro studies on orthodontic treatment [30, 32, 39, 40, 56], when simulating the application of multibracket appliance together with occlusal forces [4, 57]. Moreover, the force range of CTS that is of therapeutic significance and within the physiological range [9, 28] is broader than that of static tensile force [53], resulting in easier manipulation and investigation of magnitude-dependent effects.

A previous study showed that mechanical stress stimulates the expression of endogenous TNF-α among the other inflammatory mediators [49]. Theoretically, such production can enhance the inflammatory response in hPDLSCs, but such contribution is rather questionable for our study. This statement is based on the fact that the levels of TNF-α production by hPDLSCs are rather low. Even upon the stimulation with various bacteria the levels of TNF-α produced by hPDLSCs does not exceed 50 pg/ml [58], which is substantially lower than the concentration of exogenous TNF-α used in our experiments (10 ng/ml). Moreover, our own data show that in FBS-free media the levels of TNF-α in the conditioned media are below the detection limit of conventional ELISA, which is about a few pg/ml.

Of note, even though applying mechanical force directly on cells was widely conducted in in vitro studies, this simulation of orthodontic forces has some obvious limitations. Firstly, native paradental cells are surrounded by oriented PDL fibers when strained by tooth displacement during orthodontic tooth movement [23]. In tensioned PDL, cells are deformed within a specific three-dimensional structure, which cannot be reproduced by two-dimensional cell culture in vitro. Secondly, besides generating direct strain on matrix or cells, orthodontic forces disturb the blood flow and nerve endings, causing acute exudative inflammation and leading to a complicated biological environment [23, 59]. However, in vitro cells are cultured in an oversimplified environment, affected by the stretching alone during simulating orthodontal forces in vitro. Thirdly, due to the irreproducibility of the in vivo condition, the strain conditions of tooth displacements cannot be correlated well with the deformation condition of in vitro cell stretching; even though some researchers applied in vitro strain values based on the numerical data of PDL strains on a maxillary central incisor model [30, 56, 60–62].

In our study, we applied up to 12% CTS with the frequency of 0.1 Hz because it was the commonly used protocol for human PDL cells [37, 56, 62]. Magnitude is a critical parameter of CTS, which could bring on disparate effects on inflammatory responses, but the pattern of magnitude-dependence was not reported consistently. CTS-modulated production of inflammatory cytokines, both under basal or inflammatory stimulation environment, was observed to increase with the force magnitude [48, 49, 52] or remain no increase until a certain force magnitude level. [9, 28, 31]. Furthermore, the tolerance of PDL cells to CTS of different magnitude was shown to be decreased by periodontitis [9]. Therefore, the role of CTS magnitude was of interest for this study. 6 and 12% deformation were both identified to be within the physiological range based on their benefits to bone regeneration during orthodontic treatment [37]. For investigating inflammation regulation, 6% was commonly used within the “low force level” range, which normally exerted anti- or slightly pro-inflammatory effects in a force gradient [28, 31]. Whereas 12% might be in a “critical range”, which performed differently in different studies, generating apparent aggravation or no aggravation of inflammation [28, 49]. The loading time periods of 6- and 24 h were chosen to reflect initial stages of applied orthodontic forces [33].

The modulations of pro-inflammatory cytokines by mechanical stress have already been described in various studies. Briefly, mechanical stress with specific parameters can exert pro-inflammatory effects in human PDL cells, including up-regulation of IL-6 and IL-8. For example, the application of CTS with 10% elongation and a frequency of 1 Hz yielded an increased the level of IL-6 in primary human PDL cells within the first 2- or 6 h of strain [48]. Similar observations with an increased IL-6 have been made with the application of static tension [57], hydrostatic pressure (HP) [26], or vibration with 30 Hz [63]. Moreover, IL-8 expression was up-regulated under the application of 3 ~ 15% cyclic stretching with a frequency of 0.2 Hz after 24 h [49]. These studies indicate that human PDL cells are involved in the development of local aseptic inflammation after mechanical loading. However, our study demonstrated the CTS have no influence on the basal IL-6 and IL-8 expression. The contradictory data between our study and previous reports may be attributed to the differences in the applied strain, such as frequency and force types, various loading systems, and different culture plates.

Our data show that the expression of IL-6 in TNF-α treated hPDLSCs was inhibited by CTS at both, 6 and 12% elongation. The effects of mechanical stress under an existing inflammatory microenvironment were frequently described as magnitude-dependent [9, 31, 64], emphasizing the force threshold of aggravating periodontitis. Although the parameters in these studies involved were different, generally, CTS with higher magnitudes caused a more pronounced aggravation of inflammation whereas lower magnitudes exerted more slightly pro-inflammatory, no pro-inflammatory, or even anti-inflammatory effects in some in vitro studies [28, 29, 31, 51]. For example, Liu J et al. [9] reported that CTS with less than 8% elongation induced the minimal inflammatory response in periodontitis derived hPDLSCs. CTS with 8% elongation induced slighter enhancement of IL-6 than CTS with higher elongation. Similarly, CTS was reported to diminish IL-1β induced inflammatory response by some previous studies [28, 64]. Thus, low magnitude CTS seems to decrease IL-6 production by hPDLSCs in the inflammatory environment.

Some contradictory data were obtained on the effect of cyclic mechanical strain on IL-8 expression in hPDLSCs in the presence of TNF-α. On the one hand, TNF-α-induced gene expression of IL-8 was significantly decreased by the CTS. On the other hand, CTS enhanced TNF-α-induced IL-8 protein production. This observation suggests that the regulation of IL-8 gene expression by CTS may also occur at the post-transcriptional level. The mechanisms of such regulation in hPDLSCs are unclear to date but might involve some epigenetic factors and changes in mRNA expression. A study on human chondrocyte showed that the methylation of IL-8 promoter is decreased upon the stimulation with the pro-inflammatory cytokine IL-1β [65]. However, the existence of such an effect in hPDLSCs upon the stimulation with TNF-α should be proved. The mechanosensitive epigenetic factor was investigated in vascular endothelium. Epigenetic pathways respond to changes in blood flow and pressure in endothelial cells, with important consequences for regulating gene expression [66]. MicroRNAs (miRNAs) were reported mechanosensitive in endothelial cells, on which epigenetic modification regulates gene expression post-transcriptionally by targeting mRNA transcripts and regulating the mRNA lifetimes [66, 67]. However, the specific effects of orthodontic forces on post-transcriptional modification in the response of hPDLSCs to inflammatory stimulation need to be studied in future studies.

Our data reported that CTS upregulated the protein level of IL-8 induced by TNF-α after 24 h. The increase depended on the force magnitude. These findings suggest that, under pathological conditions, IL-8 is more sensitive to CTS than IL-6, implying the potential of IL-8 as a molecule marker for early monitoring orthodontic forces in periodontitis patients. IL-8 was reported to be elevated by mechanical forces both in human PDL cells in vitro and in animal OTM model especially in the tension area [49, 68], which is consistent with our findings. In short, the contribution of mechanical loading to periodontal inflammation cannot directly be summarized as pro- or anti-inflammatory effects. The underlying mechanism involves complex molecular signaling networks, needed further exploration.

VCAM-1 and ICAM-1 are adhesion molecules, which are usually expressed on endothelial cells and participate in the transendothelial migration of immune cells into connective tissue [69]. The expression of these molecules is upregulated by inflammatory cytokine like IL-1β and TNF-α [69]. Recent studies showed that VCAM-1 and ICAM-1 are expressed in the connective tissue cells, like gingival fibroblasts and human PDL cells, which may be related to the progression of periodontitis [70, 71]. Our study is the first which demonstrates that CTS downregulated the TNF-α-induced VCAM-1 and ICAM-1 on hPDLSCs. The biomechanical responses of adhesion molecules were widely studied on endothelial cells due to the concern of heart valve diseases. The in vitro CTS were reported that it caused an up-regulation of VCAM-1 and ICAM-1 in endothelial cells [72, 73], but the results obtained from other studies were not consistent. Breen et al. reported that the upregulation of ICAM-1 in human umbilical vein endothelial cells (HUVEC) induced by shear stress was downregulated by the addition of CTS between 4 and 12% [74]. The different effects may be attributed to the differences between application of physiological and pathological stretch [72].

## Conclusion

In conclusion, this in vitro study demonstrated that application of CTS with 6% or 12% elongation decreased the TNF-a induced gene and protein expression of IL-6, VCAM-1 and ICAM-1. CTS decreased the TNF-α induced IL-8 gene expression whereas it enhanced the protein production in a magnitude-dependent manner. Our study shows that mechanical force differentially regulates periodontitis-related inflammatory cytokines at the early stage of force application. The upregulation of IL-8 and the downregulation of IL-6 indicate that the effects of orthodontic force on inflammation could not be simply defined by pro- or anti-inflammation effects. Further investigations are needed to clarify the exact downstream effects of the cytokines on cell functions and cell behaviors. The inhibition of cell adhesion molecules suggests that mechanical sensitive mediators are involved in regulating the inflammatory processes, which provides an additional potential mechanism of the interaction between orthodontic forces and periodontitis.

## Supplementary Information

Below is the link to the electronic supplementary material.

Supplementary file1 (PDF 708 KB)
