# Supplementary material for: Cyclic tensile strain affects the response of human periodontal ligament stromal cells to tumor necrosis factor-α
Source: Clin Oral Investig. 2021 Jun 29;26(1):609–22. doi: 10.1007/s00784-021-04039-8 (PMC8791913; doi:10.1007/s00784-021-04039-8)
Supplement: Supplementary file 1 — Supplementary file1 (PDF 708 KB) [file 784_2021_4039_MOESM1_ESM.pdf]

Supplementary Material

Supplementary Figure 1

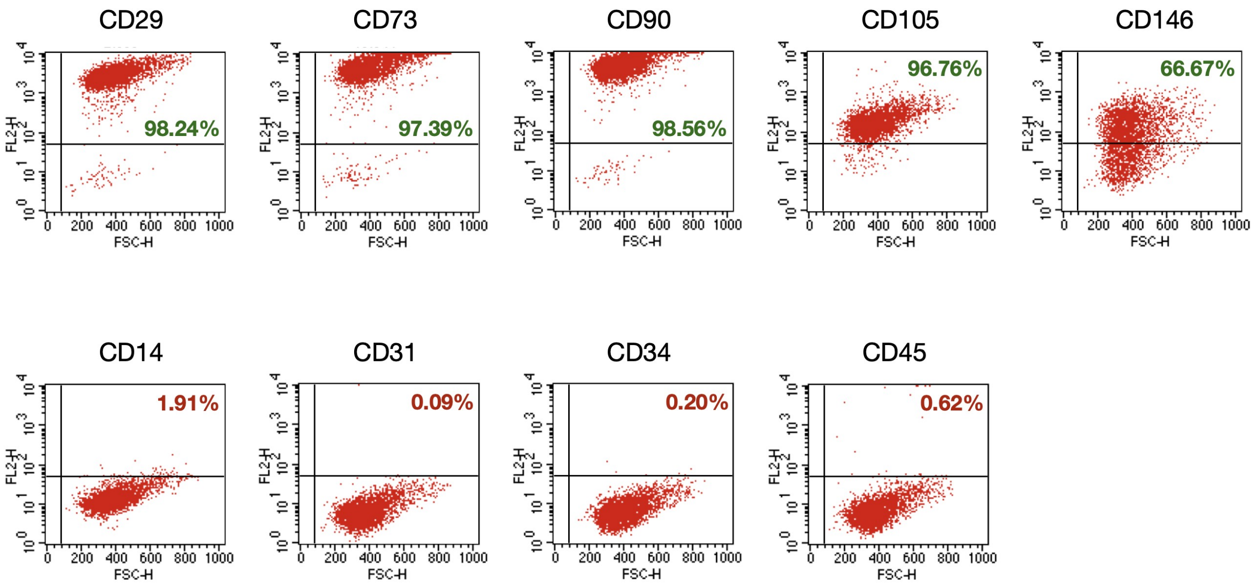

Figure S1. Representative dot plots showing the percentages of hPDLSCs which are positive for mesenchymal or hematopoietic stem cell markers.

Clinical Oral Investigation

Cyclic Tensile Strain Affects the Response of Human Periodontal Ligament Stromal Cells to Tumor Necrosis Factor-α

Zhongqi Zhao, Christian Behm, Marco Aoqi Rausch Zhiwei Tian, Xiaohui Rausch- Fan, Oleh Andrukhov

University Clinic of Dentistry, Division of Conservative Dentistry and Periodontology Sensengasse 2A, 1090 Vienna, Austria
oleh.andrukhov@meduniwien.ac.at
